# Book Reviews

**Published:** 1852-11

**Authors:** 


*Observations on the Origin, Treatment, and Cure of Asiatic Cholera and Cholera Morbus, etc.* By J. X. Chabert, M. D., Prof. of Chemistry, &c.

Allusion is made to this pamphlet in another article. Since writing that article we have been favored with a copy. The author proposes to sell the secret of a preparation which he asserts to be an infallible remedy for cholera. He affixes an M. D. to his name. Whether he has any right so to do, or not, we cannot say; but a perusal of the pamphlet will satisfy any medical reader that he is essentially a quack. We have observed several articles in a contemporary medical journal by the same author. After the publication of such a pamphlet it is to be hoped he will cease to be recognized among respectable contributors to legitimate medical literature.

---

*The Duties of Medical Men to themselves, and to their Profession. An Address before the New Hampshire Medical Faculty.* By the President, Prof. E. R. Peaslee, A. M., M. D. Published by order of the Society.

This, like all the productions of Prof. P., displays the scholar and the sound thinker. We have read the address with pleasure and profit.

